# Editorial: DNA methylation, tumor microenvironment and their effects in immunotherapy and drug resistance in thoracic tumors

**DOI:** 10.3389/fimmu.2024.1357278

**Published:** 2024-01-15

**Authors:** Limeng Qu, Shirong Ding, Qian Long, Shaoquan Zheng, Zhe-Sheng Chen, Wenjun Yi

**Affiliations:** ^1^ Department of General Surgery, The Second Xiangya Hospital, Central South University, Changsha, Hunan, China; ^2^ Department of Oncology, The Second Xiangya Hospital, Central South University, Changsha, Hunan, China; ^3^ Clinical Research Center For Breast Disease In Hunan Province, Changsha, China; ^4^ Department of Breast Surgery, Breast Disease Center, The First Affiliated Hospital, Sun Yat-sen University, Guangzhou, China; ^5^ Department of Pharmaceutical Sciences, College of Pharmacy and Health Sciences, St John’s University, Queens, NY, United States

**Keywords:** DNA methylation, tumor microenvironment, immunotherapy, drug resistance, thoracic tumors

Thoracic tumors, such as lung cancer and breast cancer, are major contributors to cancer-related fatalities, and their incidence is rapidly escalating (1). Currently, clinical treatments for thoracic tumors, including surgery, chemotherapy, and molecular targeted therapy, have limitations. While immunotherapy has made significant advancements in the treatment of thoracic tumors, a notable proportion of patients still experience drug resistance. Consequently, the quest for novel, safe, and effective therapies to mitigate the progression of thoracic malignant tumors and extend patient survival remains an urgent clinical challenge. DNA methylation is a common epigenetic modification that significantly regulates gene expression, cell differentiation, genome stability, and development (2). The tumor microenvironment (TME) encompasses the surrounding environment where tumor cells thrive and multiply. It not only provides the necessary resources and energy for tumor cell growth, proliferation, invasion, and metastasis but also plays a regulatory role in tumor progression by impacting the responsiveness of tumor cells to immunotherapy (3). Thus, conducting in-depth research on the underlying mechanisms through which the TME regulates tumor progression and identifying effective targets and markers for treating malignant tumors hold great significance. This Research Topic, titled “ *DNA methylation, Tumor Microenvironment and their Effects in Immunotherapy and Drug Resistance in Thoracic Tumors*,” was curated by four guest editors and consists of a collection of eight articles, including three reviews and five original research studies. These articles provide new perspectives on the involvement of DNA methylation and the TME in immunotherapy and drug resistance in thoracic tumors. They also discuss potential strategies to overcome these challenges, making valuable contributions to the field.

Lung cancer remains the leading cause of cancer-related deaths worldwide, with lung adenocarcinoma (LUAD) being a prevalent subtype of non-small cell lung cancer, accounting for approximately 45% of lung cancer cases (4, 5). In recent years, the advent of single-cell RNA sequencing (scRNA-seq) has revolutionized the study of the TME and mechanisms underlying tumor progression (6). Xue et al. have provided a comprehensive overview of the latest research on scRNA-seq in lung cancer, shedding light on cellular developmental trajectories, phenotypic remodeling, and cellular interactions during tumor progression. Considering the molecular heterogeneity of LUAD, Wu et al. conducted scRNA-seq analysis and developed a prognostic model based on the Malignancy-Related Risk Score (MRRS). This model enables efficient prediction of the prognosis for patients with LUAD. Moreover, further investigations revealed the association of MRRS with oncogenic pathways, gene mutations, and immune function, providing insights into the potential mechanisms driving LUAD progression. Significantly, considering the pivotal role of mast cells in the tumor immune microenvironment and the conflicting findings in current studies regarding their impact on the prognosis of LUAD, Zhang et al. established a prognostic tool consisting of nine mast cell-related genes (MRGs) that effectively predicted immunotherapy response in LUAD patients by analyzing scRNA-seq and bulk RNA-seq data.

MiRNAs are small non-coding RNAs that regulate gene expression at the post-transcriptional level and play an important role in the TME by inhibiting gene expression through RNA interference (7). Theresa Kordaß et al. discovered that miR-1285-5p, miR-155-5p, and miR-3134 exhibited the most potent inhibitory effect on NT5E expression. Conversely, miR-134-3p, miR-6859-3p, miR-6514-3p, and miR-224-3p were found to strongly promote the expression of NT5E. These findings highlight the potential of these miRNAs as therapeutic agents or targets for clinical applications. It has been shown that miRNAs can be used to improve oncolytic viruses (OVs) for better viral oncolysis, tumor suppression, and immunomodulation (8). St-Cyr et al. systematically reviewed the ways in which miRNAs synergistically enhance OV immunotherapy, demonstrating the emerging synergistic platform constituted by the combination of MiRNA therapeutics and OVs, which provides a solid basis for enhancing immunomodulation in TME. Small nucleolar RNA host genes (SNHGs) are a group of genes that can transcribe long noncoding RNA SNHGs (lncSNHGs) and further process them into small nucleolar RNAs (SnoRNAs). To better understand how the transcripts of SNHGs are involved in tumorigenesis from an immune perspective, Xiao et al. systematically sorted out the expression of snoRNAs and lncSNHGs in different immune cell types and summarized the typical and atypical mechanisms by which snoRNAs regulate antitumor immunity. In addition, the authors reviewed the roles and mechanisms of lncSNHGs in the regulation of immune cell functions, indicating that snoRNAs and lncSNHGs may be promising biomarkers and therapeutic targets for cancer immunotherapy.

This Research Topic also contributes to a more comprehensive understanding of TME, immunotherapy and drug resistance in thyroid and breast cancer. Medullary thyroid carcinoma (MTC) is less differentiated and more aggressive than other types. Weng et al. explored the gene expression pattern of MTC and constructed a TF-mRNA-miRNA regulatory network consisting of 5 TFs, 9 Hub genes, and 13 miRNAs by comprehensive analysis, which suggested that TFs, Hub genes, and miRNAs may serve as oncogenic biomarkers and therapeutic targets for MTC. Triple-negative breast cancer (TNBC), as a subtype of breast cancer with poor prognosis, TME plays a key role in its tumor progression. Li et al. included 1,604 studies for bibliometric analysis in order to explore the trends and research areas of TME in TNBC, thus providing insights into the research trends related to TME studies in TNBC globally, suggesting that there is a bright future for TME-related clinical research in TNBC.

In summary, this Research Topic offers a comprehensive analysis of eight articles that shed light on the relationship between DNA methylation, the TME, and their influence on immunotherapy and drug resistance in thoracic tumors. These articles approach the topic from different perspectives, providing valuable insights and potential therapeutic strategies. Unfortunately, this Research Topic has less to say about DNA methylation for tumor immunotherapy, which is a worthy direction for research. Basic and clinical research based on DNA methylation and TME as a target will promote the progress of tumor mechanism research, optimize the therapeutic regimen for tumor patients, and ultimately improve the long-term prognosis of tumor patients. We hope that the findings presented in this Research Topic will provide a solid foundation and the right direction for future research to maximize the benefits to oncology patients.

## Author contributions

LQ: Writing – original draft. SD: Writing – original draft. QL: Writing – original draft, Writing – review & editing. SZ: Writing – review & editing. Z-SC: Writing – review & editing. WY: Writing – review & editing.
